# Modelling the neuropathological consequences of HIV vaccines that confer partial protection

**DOI:** 10.1186/1742-4690-9-S2-P30

**Published:** 2012-09-13

**Authors:** D Ferguson, S Clarke, C Ham, A Das, B Berkhout, A Meiser, S Patterson, N Berry, N Almond

**Affiliations:** 1 National Institute of Biological Standards and Control - HPA, Potters Bar, UK; 2 Academic Medical Centre, University of Amsterdam, Amsterdam, the Netherlands; 3 Imperial College, London, UK

## Background

Effective management of peripheral viral loads with anti-retroviral drugs can delay or halt CD4 cell loss for decades. However, patients still face the potential of developing significant HIV-1 associated neurocognitive disorders (HAND). Challenges obtaining relevant clinical samples limit the detailed investigation of the aetiology and neuropathology of HAND and therefore we do not understand the impact of vaccines that confer partial protection on long term neuropathology.

## Methods

We are using immunohistochemical analysis of brain sections from the experimental infection of macaques with SIV to model neuropathology in situations where peripheral viral loads are under effective control.

## Results

We have established that chronic infection with attenuated SIV, where peripheral viral loads are below detection, still results in pathological changes (astrogliosis, microglial activation, viral persistence). We have now extended these studies using a unique conditional live attenuated, doxycycline dependent virus, SIVrtTA. Removal of doxycycline 3 weeks after infection, at the end of the primary viremia results in detectable neuropathological changes in astrocytes, oligodendrocytes, microglia and perivascular macrophage 40 weeks later.

We are also investigating the effects of vaccines that confer partial protection on the neuropathology of wild-type SIV infection. A vaccine study using SIV Gag based vaccines comprising three primes with rDNAgag followed by a rAdgag boost resulted in significant delay in acquisition of SIVmac251 administered by low-dose intra-rectal challenge. Furthermore, there was a significant blunting of the primary viremia, but no significant suppression of set-point viral loads compared with naive challenge controls. We are determining the impact of vaccination on the frequency of virus infected cells in the brain collected 20-30 weeks after infection and also comparing the frequency and intensity of neuropathological changes in infected vaccinated and control macaques.

## Conclusion

These data will enable us to establish the likely neurological benefit of vaccines that do not provide protection against detectable infection.

